# Supplementary material for: Validation of a refined protocol for mouse oral glucose tolerance testing without gavage
Source: bioRxiv. 2024 Sep 19:2024.09.13.612859. Preprint. [Version 1] doi: 10.1101/2024.09.13.612859 (PMC11429937; doi:10.1101/2024.09.13.612859)
Supplement: Supplement 1 [file NIHPP2024.09.13.612859v1-supplement-1.pdf]

bioRxiv preprint doi: https://doi.org/10.1101/2024.09.13.612859; this version posted September 19, 2024. The copyright holder for this preprint (which was not certified by peer review) is the author/funder, who has granted bioRxiv a license to display the preprint in perpetuity. It is made available under aCC-BY 4.0 International license.

Supplementary Figures

Supplementary Figure 1:

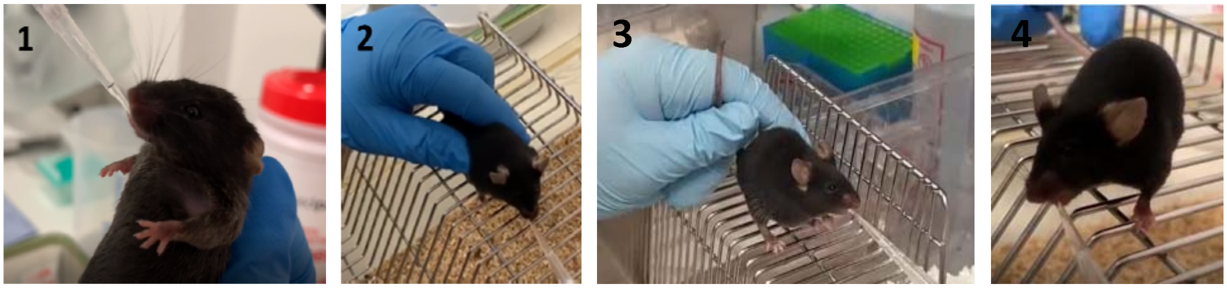

Supplementary Figure 1: Habituation of C57BL/6J mice to micropipette-guided dosing with chocolate-flavoured glucose (40% solution with flavouring).

1: Mice were restrained via scruff and the pipette containing the glucose solution was presented at a horizontal angle.

2: Mice were gently restrained atop the hopper with a light grip on their tail and above their hind legs, and the pipette was once again presented at a horizontal angle.

3: Mice were held gently by the tail and the pipette was presented as before.

4: Mice were placed atop the hopper and drank from the pipette without restraint.

Supplementary Figure 2:

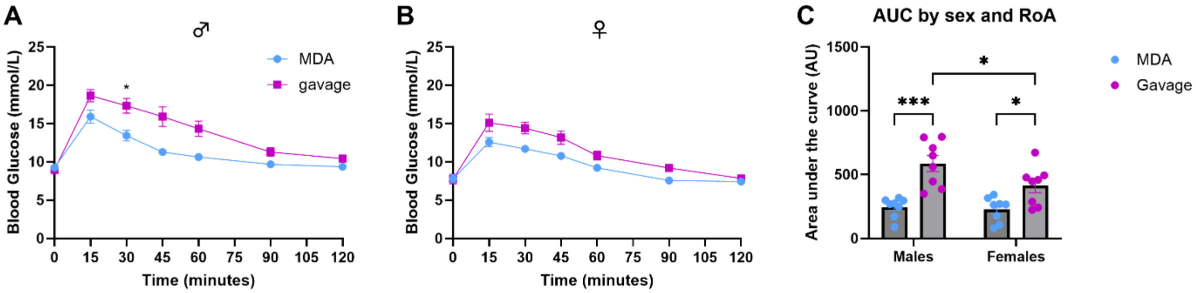

Supplementary figure 2: MDA-glucose dosing produced a glucose clearance profile comparable in shape to that of gavage-glucose dosing in an oral glucose tolerance test (oGTT) in lean C57BL/6J mice (prior to introduction of the high-fat diet). Using a within-subjects randomised cross-over design with order of route of administration balanced, male and female C67BL/6J mice (8 males, 8 females) were subjected to two oGTTs one-week apart prior to the introduction of a high-fat diet (see **figure 4**). For each oGTT, animals were dosed orally with 2.5g glucose/kg body weight of flavoured glucose solution via gavage (magenta) or micropipette-guided drug administration (MDA; blue) after a basal blood glucose measurement (tail prick) at 0 minutes. Blood glucose was then measured at regular intervals for up to 120 minutes. For both sexes (**A-C**) there was a statistically larger baseline subtracted area under the curve (AUC; **C**) in the gavage-oGTT compared to the MDA-oGTT, and a sex difference in the AUC of the gavage, but not the MDA-oGTT. AUC data analysed by two-way repeated measures ANOVA for effects of sex ($F_{(1,\ 14)}$ = 4.13, $p_{(sex)}$ = 0.06), and route of administration (RoA; $F_{(1,\ 14)}$ = 31.9, $p_{(RoA)}$ <0.0001), with multiple comparisons (* p<0.05; *** p<0.001). Data are presented as mean ± SEM.

bioRxiv preprint doi: https://doi.org/10.1101/2024.09.13.612859; this version posted September 19, 2024. The copyright holder for this preprint (which was not certified by peer review) is the author/funder, who has granted bioRxiv a license to display the preprint in perpetuity. It is made available under aCC-BY 4.0 International license.

Supplementary Tables

Supplementary table 1: Effect of sex and route of administration (RoA) on plasma corticosterone.

Data analysed by 2-way repeated measures analysis of variance (RM-ANOVA) for sex, RoA interaction. Bolded values indicate statistical significance (*p*<0.05).

**Figure 2**	N	Sex	RoA	RoA x Sex
C57BL/6Js	18 (11m, 7f)	$F_{(1,14)} = 1.98$ $p = 0.18$ (ns)	**$F_{(1,14)} = 30.9$ $p < 0.0001$ (****)**	$F_{(1,14)} = 0.014$ $p = 0.91$ (ns)

Supplementary table 2: Comparisons of C57BL/6J and C57BL/6N data.

Glucose tolerance test (GTT) curve data for each strain after a randomised crossover, where each mouse was given both a MDA-oGTT and a gavage-oGTT one week apart, with the order of dosing routes randomised. Data were analysed by 3-way repeated measures analysis of variance (RM-ANOVA) for time, sex, and route of administration (RoA) interaction with Tukey's multiple comparisons test. Area under curve (AUC), basal blood glucose (BG), and peak size data for each strain were analysed by 2-way RM-ANOVA for sex and RoA interaction. AUC and peak size strain comparisons were analysed by 2-way RM-ANOVA for RoA, strain interaction. Bolded values indicate statistical significance (*p*<0.05). ns = not statistically significant (*p*>0.05).

**C57BL/6J mice (Figure 3A-C; N = 14 [8 male and 6 female])**									
	Sex	RoA	Time x RoA	Time x Sex	RoA x Sex	Time x RoA x Sex	Sub-strain	Sex x sub-strain	RoA x sub-strain
GTT curve	$F_{(1,14)} = 1.24$ $p = 0.28$ (ns)	$F_{(0.4,5.2)} = 3.56$ $p = 0.11$ (ns)	$F_{(2.9,27.3)} = 1.30$ $p = 0.30$ (ns)	$F_{(6,84)} = 0.34$ $p = 0.92$ (ns)	$F_{(1,14)} = 0.11$ $p = 0.75$ (ns)	$F_{(6,56)} = 1.28$ $p = 0.28$ (ns)	-	-	-
Basal blood glucose	$F_{(1,14)} = 1.91$ $p = 0.19$ (ns)	$F_{(1,10)} = 0.37$ $p = 0.56$ (ns)	-	-	$F_{(1,10)} = 0.0003$ $p = 0.99$ (ns)	-	-	-	-

bioRxiv preprint doi: https://doi.org/10.1101/2024.09.13.612859; this version posted September 19, 2024. The copyright holder for this preprint (which was not certified by peer review) is the author/funder, who has granted bioRxiv a license to display the preprint in perpetuity. It is made available under aCC-BY 4.0 International license.

Baseline subtracted AUC	$F_{(1,12)}$ = 0.13 p = 0.72 (ns)	**$F_{(1,12)}$ = 6.29 p = 0.028 (*)**	-	-	$F_{(1,12)}$ = 0.15 p = 0.70 (ns)	-	-	-	-
Peak size	$F_{(1,12)}$ = 0.085 p = 0.78 (ns)	$F_{(1,12)}$ = 0.88 p = 0.37 (ns)	-	-	$F_{(1,12)}$ = 0.019 p = 0.89 (ns)	-	-	-	-
**C57BL/6N mice (Figure 3D-F; N = 16 [8 male and 8 female])**									
	Sex	RoA	Time x RoA	Time x Sex	RoA x Sex	Time x RoA x Sex	Sub-strain	Sex x sub-strain	RoA x sub-strain
GTT curve	**$F_{(1,14)}$ = 28.0 p = 0.0001 (***)**	$F_{(0.5,7.4)}$ = 2.55 p = 0.15 (ns)	$F_{(3.0,41.6)}$ = 0.62 p = 0.60 (ns)	$F_{(6.84)}$ = 2.01 p = 0.073 (ns)	$F_{(1,14)}$ = 0.35 p = 0.56 (ns)	$F_{(6.84)}$ = 0.38 p = 0.89 (ns)	-	-	-
Basal blood glucose	$F_{(1,14)}$ = 2.43 p = 0.14 (ns)	$F_{(1,14)}$ = 1.38 p = 0.26 (ns)	-	-	$F_{(1,14)}$ = 0.038 p = 0.85 (ns)	-	-	-	-
Baseline subtracted AUC	$F_{(1,14)}$ = 0.93 p = 0.35 (ns)	$F_{(1,14)}$ = 0.25 p = 0.62 (ns)	-	-	$F_{(1,14)}$ = 0.21 p = 0.65 (ns)	-	-	-	-
Peak size	$F_{(1,14)}$ = 1.10 p = 0.31 (ns)	$F_{(1,14)}$ = 0.078 p = 0.78 (ns)	-	-	$F_{(1,14)}$ = $1.50e^{-030}$ $p > 0.99$ (ns)	-	-	-	-
**Sub-strain comparison (Figure 3G and H; N = 14 C57BL/6J mice [8 male and 6 female] and N = 16 [8 male and 8 female] C57BL/6N mice)**									
	Sex	RoA	Time x RoA	Time x Sex	RoA x Sex	Time x RoA x Sex	Sub-strain	Sex x sub-strain	RoA x sub-strain
Basal blood glucose	$F_{(1, 26)}$ = 3.51 p =	**-**	-	-	-	-	$F_{(1, 26)}$ = 0.46 p =	$F_{(1, 26)}$ = 0.019 p =	-

bioRxiv preprint doi: https://doi.org/10.1101/2024.09.13.612859; this version posted September 19, 2024. The copyright holder for this preprint (which was not certified by peer review) is the author/funder, who has granted bioRxiv a license to display the preprint in perpetuity. It is made available under aCC-BY 4.0 International license.

	0.072						0.504	0.89	
Baseline subtracted AUC	-	**$F_{(1,28)}$ = 4.66 *p* = 0.040 (*)**	-	-	-	-	**$F_{(1,28)}$ = 21.0 *p* <0.0001 (****)**	-	$F_{(1,28)}$ = 2.05 *p* = 0.16 (ns)
Peak size	-	$F_{(1,28)}$ = 1.14 *p* = 0.30 (ns)	-	-	-	-	**$F_{(1,28)}$ = 30.9 *p* <0.0001 (****)**	-	$F_{(1,28)}$ = 0.63 *p* = 0.44 (ns)

Supplementary table 3a: HFD data

C57BL/6J mice (n= 8 males, 7 females) were given two oGTTs (MDA-oGTT and gavage-oGTT one week apart) in a randomised crossover before and after a 16-week high fat diet (HFD). Lean and HFD GTT data were analysed by 3-way repeated measures analysis of variance (RM-ANOVA) for time, sex, route of administration (RoA) interaction with Tukey's multiple comparison test. Comparisons of area under curve (AUC), basal blood glucose (BG) and peak size between diets were analysed by 3-way RM-ANOVA for sex, RoA, diet interaction with Tukey's multiple comparisons test. Bolded values indicate statistical significance (*p*<0.05).

**C57BL/6J mice (Figure 4A-F; N = 15 [8 male and 7 female])**										
	Sex	RoA	Diet	Time x RoA	Time x Sex	RoA x Sex	RoA x Diet	Diet x sex	Time x RoA x Sex	Diet x RoA x Sex
Lean GTT curve	**$F_{(1,14)}$ = 15.1 *p* = 0.0017 (**)**	**$F_{(0.5,7.2)}$ = 34.2 *p* = 0.0015 (**)**	-	**$F_{(2.7,37.8)}$ = 7.22 *p* = 0.0009 (***)**	**$F_{(6,84)}$ = 2.46 *p* = 0.031 (*)**	$F_{(1,14)}$ = 1.54 *p* = 0.24 (ns)	-	-	$F_{(6,84)}$ = 0.98 *p* = 0.44 (ns)	-
HFD GTT curve	**$F_{(1,13)}$ = 8.92 *p* = 0.011 (*)**	**$F_{(0.5,6.6)}$ = 10.6 *p* = 0.024 (*)**	-	**$F_{(3.6,46.3)}$ = 7.81 *p* = 0.0001 (***)**	$F_{(6,78)}$ = 2.09 *p* = 0.064 (ns)	$F_{(1,13)}$ = 0.37 *p* = 0.56 (ns)	-	-	**$F_{(6,78)}$ = 3.41 *p* = 0.0049 (**)**	-
Basal subtracted AUCs	$F_{(1,14)}$ = 0.92 *p* = 0.35 (ns)	**$F_{(1,14)}$ = 68.1 *p* <0.0001 (****)**	$F_{(1,14)}$ = 1.82 *p* = 0.20 (ns)	-	-	$F_{(1,14)}$ = 0.23 *p* = 0.64 (ns)	$F_{(1,12)}$ = 0.52 *p* = 0.48 (ns)	$F_{(1,14)}$ = 2.37 *p* = 0.15 (ns)	-	$F_{(1,12)}$ = 3.03 *p* = 0.11 (ns)

bioRxiv preprint doi: https://doi.org/10.1101/2024.09.13.612859; this version posted September 19, 2024. The copyright holder for this preprint (which was not certified by peer review) is the author/funder, who has granted bioRxiv a license to display the preprint in perpetuity. It is made available under aCC-BY 4.0 International license.

Basal blood glucose	**$F_{(1,14)}$ = 16.6 *p* = 0.0011 (**)**	$F_{(1,14)}$ = 0.16 *p* = 0.69 (ns)	**$F_{(1,14)}$ = 33.6 *p* <0.0001 (****)**	-	-	$F_{(1,14)}$ = $4.1x10^{-4}$ *p* = 0.98 (ns)	$F_{(1,12)}$ = 0.015 *p* = 0.90 (ns)	$F_{(1,14)}$ = 0.82 *p* = 0.38 (ns)	-	$F_{(1,12)}$ = 0.15 *p* = 0.71 (ns)
Peak size	$F_{(1,54)}$ = 0.20 *p* = 0.66 (ns)	**$F_{(1,54)}$ = 16.3 *p* = 0.0002 (***)**	**$F_{(1,54)}$ = 7.26 *p* = 0.0094 (**)**	-	-	$F_{(1,54)}$ = 1.26 *p* = 0.27 (ns)	$F_{(1,54)}$ = 1.26 *p* = 0.27 (ns)	**$F_{(1,54)}$ = 11.7 *p* = 0.0012 (**)**	-	$F_{(1,54)}$ = 2.30 *p* = 0.14 (ns)

Supplementary table 3b: Vanderbilt MMPC-Live C57BL/6J mice

C57BL/6J mice were given two oGTTs (MDA-oGTT and gavage-oGTT one week apart) in a randomised crossover. Glucose tolerance test (GTT) curve data for each sex were analysed by 3-way repeated measures analysis of variance (RM-ANOVA) for time, sex, and route of administration (RoA) interaction with Tukey's multiple comparisons test. Baseline-corrected area under curve (AUC) and peak size data were analysed by 2-way RM-ANOVA for sex and RoA interaction. Bolded values indicate statistical significance (*p*<0.05).

**Vanderbilt C57BL/6J mice (Figure 3A-C; N = 16 [8 male and 8 female])**						
	Sex	RoA	Time x RoA	Time x Sex	RoA x Sex	Time x RoA x Sex
C57BL/6J GTT curve	$F_{(1,14)}$ = 0.34 *p* = 0.57 (ns)	**$F_{(0.4,5.2)}$ = 20.4 *p* = 0.012 (*)**	**$F_{(2.6,36.2)}$ = 7.76 *p* = 0.0007 (***)**	$F_{(6, 84)}$ = 1.22 *p* = 0.30 (ns)	$F_{(1,14)}$ = 2.86 *p* = 0.11 (ns)	**$F_{(6,84)}$ = 3.37 *p* = 0.005 (**)**
Baseline subtracted AUC	$F_{(1,14)}$ = 1.25 *p* = 0.28 (ns)	**$F_{(1,14)}$ = 18.9 *p* = 0.0007 (***)**	-	-	**$F_{(1,14)}$ = 4.83 *p* = 0.045 (*)**	-
Peak size	$F_{(1,14)}$ = 8.12 *p* = 0.013 (*)	**$F_{(1,14)}$ = 9.21 *p* = 0.009 (**)**	-	-	$F_{(1,14)}$ = 2.67 *p* = 0.12 (ns)	-
